# Modeling and Fusing the Uncertainty of FMEA Experts Using an Entropy-Like Measure with an Application in Fault Evaluation of Aircraft Turbine Rotor Blades

**DOI:** 10.3390/e20110864

**Published:** 2018-11-09

**Authors:** Xuelian Zhou, Yongchuan Tang

**Affiliations:** 1 School of Computer and Information Science, Southwest University, Chongqing 400715, China; 2 School of Electronics and Information, Northwestern Polytechnical University, Xi’an 710072, China

**Keywords:** failure mode and effects analysis (FMEA), risk priority number (RPN), dempster–shafer evidence theory (DST), risk management, uncertainty measure, ambiguity measure (AM)

## Abstract

As a typical tool of risk analysis in practical engineering, failure mode and effects analysis (FMEA) theory is a well known method for risk prediction and prevention. However, how to quantify the uncertainty of the subjective assessments from FMEA experts and aggregate the corresponding uncertainty to the classical FMEA approach still needs further study. In this paper, we argue that the subjective assessments of FMEA experts can be adopted to model the weight of each FMEA expert, which can be regarded as a data-driven method for ambiguity information modeling in FMEA method. Based on this new perspective, a modified FMEA approach is proposed, where the subjective uncertainty of FMEA experts is handled in the framework of Dempster–Shafer evidence theory (DST). In the improved FMEA approach, the ambiguity measure (AM) which is an entropy-like uncertainty measure in DST framework is applied to quantify the uncertainty degree of each FMEA expert. Then, the classical risk priority number (RPN) model is improved by aggregating an AM-based weight factor into the RPN function. A case study based on the new RPN model in aircraft turbine rotor blades verifies the applicable and useful of the proposed FMEA approach.

## 1. Introduction

Failure of risk management in a complex system or a key component may lead to a total disaster [1]. Risk modeling and analysis is a hot topic in practical applications such as complex networks [2], human reliability analysis [3], maintenance of complex systems [4] and so on. Risk management methods in real applications include fault diagnosis [5,6], fault detection and isolation [7], system condition monitoring [4,8], uncertainty quantification [9] and so on. As a typical theory for modeling and processing risk analysis, failure mode and effects analysis (FMEA) theory is widely used in dealing with subjective and objective risk assessments simultaneously [10,11,12]. In FMEA approach, a FMEA item is judged by experts with subjective evaluation. The classical risk priority number (RPN) model, which is based on the subjective assessment of FMEA experts, is sometimes not that efficient for a variety of practical applications [13,14,15]. This paper proposes an improved RPN method by considering the relative importance of each FMEA member to contribute a more accurate method in uncertainty modeling and fusion of FMEA experts’ subjective evaluation.

Dempster–Shafer evidence theory (DST) [16,17,18,19], also known as the belief functions theory [20,21,22], is effective in uncertain information processing such as pattern recognition [23,24], target identification [25], risk analysis [26], controller design [27], community detection [28], and many other practical applications in the areas of information fusion [29,30,31]. The method of quantifying the uncertain degree of the body of evidence before applying information fusion attracts much attention. The approximate entropy-based uncertainty measure is a typical way for quantification of uncertain information in real applications including clinical signal analysis and processing [32,33], the graph of networks [34,35,36] and so on [37,38,39]. Similarly, in DST framework, some uncertainty measures are also approximate entropies, such as the measure of aggregate uncertainty (AU) [40], the ambiguity measure (AM) [41], the Deng entropy [42,43] and so on [44,45,46]. Other uncertainty measure in DST framework includes the belief intervals-based total uncertainty measure [47] and so on [48]. All these uncertainty measures have some advantages in practical applications such as sensor data fusion [49] and decision making [50]. As a typical entropy-like uncertainty measure in DST framework, AM has some advantages in comparison with other uncertainty measures. For example, AM satisfies all five requirements for AU measures including probability consistency, set consistency, subadditivity, additivity and so on. Based on this important feature for uncertain information processing, AM is chosen to quantify the uncertainty of FMEA experts’ evaluations.

To overcome some shortages of the classical FMEA theory, many studies focus on combining DST with FMEA to design applicable and useful risk analysis approaches. In [51,52,53], DST is introduced to fuse the belief structure of uncertain assessments from FMEA experts. The hybrid method based on DST and other methods, such as fuzzy sets theory, is also popular among researchers [54,55]. The international standard ISO 31000 [56] is also introduced to improve the FMEA method for risk identification, evaluation and control [57]. However, none of the aforementioned methods model the relative importance among different FMEA experts by considering the assessment data from the experts themselves. In other words, the relative importance of different FMEA experts in the aforementioned methods is all directly based on subjective assessments, which cannot be accurate in some cases. We argue that the assessment information itself implicates uncertainty of a FMEA expert, which should be considered when modeling the relative importance of a FMEA expert. Based on this perception, an improved RPN model is designed to model the weight of a FMEA member with respect to all the FMEA experts in a typical FMEA team.

This rest of this paper is organized as follows. Some preliminaries are introduced in Section 2. In Section 3, a new FMEA approach, which is based on the new AM-based RPN model, is proposed. Then, the proposed method is applied to a case study in Section 4. Section 5 draws the conclusion as well as shows the directions of future work.

## 2. Preliminaries

### 2.1. Dempster–Shafer Evidence Theory

A brief introduction to Dempster–Shafer evidence theory [16,17] related to the following research in this paper is presented in this section.

**Definition** **1.** *Define that $\Omega =\left\{\theta_{1},\theta_{2},\ldots ,\theta_{i},\ldots ,\theta_{N}\right\}$ is a nonempty set with N mutually exclusive and exhaustive events,* Ω *is the frame of discernment (FOD). The power set* of Ω *consists of $2^{N}$ elements denoted as follows:*

(1) $$2^{\Omega }=\left\{\begin{array}{c} \emptyset ,\left\{\theta_{1}\right\},\left\{\theta_{2}\right\},\ldots ,\left\{\theta_{N}\right\},\left\{\theta_{1},\theta_{2}\right\},\\ \ldots ,\left\{\theta_{1},\theta_{2},\ldots ,\theta_{i}\right\},\ldots ,\Omega \end{array}\right\}.$$

**Definition** **2.** *A mass function m is a mapping from the power set $2^{\Omega }$ to the interval [0,1]. m satisfies:*

(2) $$m\left(\emptyset \right)=0,\;\sum_{A\in \Omega }m\left(A\right)=1.$$

*If $m\left(A\right)>0$, then A is called a focal element. $m\left(A\right)$ indicates the support degree of the evidence on the proposition A.*

### 2.2. Failure Mode and Effects Analysis

Failure mode and effects analysis (FMEA) is a method for risk identification, prevention and management. Applying FMEA method in practical applications is a series of activities including risk identification by a FMEA team, risk assessment by each FMEA expert, ranking the priorities of FMEA items, risk control and management based on the priority of each FMEA item, and the related work in the aforementioned processes. A FMEA item is the result of risk identification with respect to a potential risk or possible hazard in a system or process. For different processes or purposes, FMEA has different classes, e.g., DFMEA for product design, SFMEA for system management, and PFMEA for process management.One of the key issues when applying FMEA method is calculating the risk priorities of different FMEA items based on the risk priority number (RPN) model.

**Definition** **3.** *In FMEA, the risk priority number (RPN) is defined as follows [12,58]:*

(3) RPN=O×S×D,

*where O is the probability of the occurrence of a FMEA item, S is the severity degree if a failure happens assessed by FMEA experts, and D is the probability of a potential FMEA item can be detected.*

Generally, the value of RPN is ranging from 1 to 10, which means a risk factor is divided into 10 ranking levels in FMEA method [51,52,53,54,58].

### 2.3. Ambiguity Measure

As a typical entropy-like uncertainty measure, ambiguity measure (AM) is proposed by Jousselme et al. [41], AM satisfies the properties and features of AU [40].

**Definition** **4.** *AM is defined as follows [41]:*

(4) $$AM\left(m\right)=-\sum_{x\in X}BetP_{m}\left(x\right)\log_{2}\left(BetP_{m}\left(x\right)\right),$$

*where $BetP_{m}$ is the pignistic probability distribution of the mass function m [20], denoted as follows:*

(5) $$BetP_{m}\left(A\right)=\sum_{B\subseteq X}m\left(B\right)\frac{\left|A\cap B\right|}{\left|A\right|},$$

*where $\left|A\right|$ means the cardinality of the set A.*

## 3. The Ambiguity Measure-Based FMEA Approach

An improved FMEA approach based on the AM-based RPN model is presented in this section. The uncertainty of FMEA experts, which is represented by the corresponding assessments, is modeled by AM in DST framework. The relative importance among different FMEA experts isconsidered according to the AM-based RPN model in the new method.

### 3.1. The New RPN Model in DST Framework

**Figure 1:**
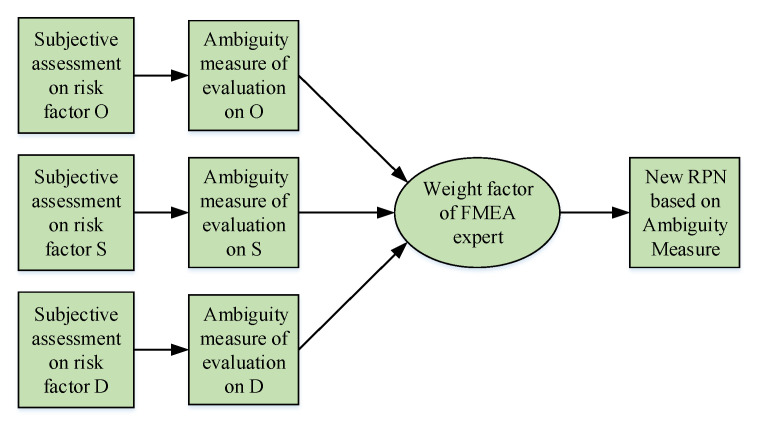
The framework of the new RPN based on the AM.

How to fuse the relative importance of different FMEA experts in the process of fusing the related assessments is still an open issue. To handle this issue, the subjective assessments of each FMEA experts on each FMEA item is analyzed and modeled as the corresponding weight factor of each FMEA expert. The block diagram showing the idea of the new RPN is presented in Figure 1, where the subjective assessment of FMEA experts on risk factor *O*, *S* and *D* is quantified based on the AM in DST framework. The relative weight of each FMEA expert is based on the proportion of its AM value with respect to the sum of all AM values. Finally, the weight factor consisting of subjective assessments of FMEA experts can be fused in the new RPN. Definition 5 presents the function of the AM-based RPN method.

**Definition** **5.** *Assume that n (n≥1) independent FMEA experts assess the FMEA items with RPN values in a FMEA team; the AM-based RPN denoted as $RPN_{am}$ is defined as follows:*

(6) $$RPN_{am}=\sum_{i=1}^{n}\frac{w\left(e_{i}\right)}{\sum_{i=1}^{n}w\left(e_{i}\right)}O_{i}S_{i}D_{i}.$$

*where $w\left(e_{i}\right)$ is weight factor of the ith FMEA expert, which is based on AM, $w\left(e_{i}\right)$ is defined as follows:*

(7) $$w\left(e_{i}\right)=AM\left(O_{i}\right)+AM\left(S_{i}\right)+AM\left(D_{i}\right),$$

*where $AM\left(\cdot \right)$ is ambiguity degree of FMEA expert, and $O_{i}$, $S_{i}$ and $D_{i}$ are the assessed rating values for each risk factor O, S and D by the ith FMEA expert.*

With Equation (4), the AM value of risk factors by the *i*th FMEA expert is defined as follows:

(8) $$\begin{array}{c} AM\left(O_{i}\right)=-\sum_{O_{i}\in A\subseteq X}BetP_{m}\left(A\right)\log_{2}\left(BetP_{m}\left(A\right)\right),\\ AM\left(S_{i}\right)=-\sum_{S_{i}\in A\subseteq X}BetP_{m}\left(A\right)\log_{2}\left(BetP_{m}\left(A\right)\right),\\ AM\left(D_{i}\right)=-\sum_{D_{i}\in A\subseteq X}BetP_{m}\left(A\right)\log_{2}\left(BetP_{m}\left(A\right)\right), \end{array}$$

where *A* is the proposition related to the assessment of the risk factor, $X=\left\{O,S,D\right\}$ is the FOD of risk factors, and $BetP_{m}\left(A\right)$ is the pignistic probability distribution of $m\left(A\right)$. The fused rating values of $O_{i}$, $S_{i}$ and $D_{i}$ assessed by the *i*th expert is defined as follows:

(9) $$\begin{array}{c} O_{i}=\sum_{j=1}^{10}R_{j}m_{j}\left(O_{i}\right),\\ S_{i}=\sum_{j=1}^{10}R_{j}m_{j}\left(S_{i}\right),\\ D_{i}=\sum_{j=1}^{10}R_{j}m_{j}\left(D_{i}\right), \end{array}$$

where *j* = (1, 2, …, 10), $R_{j}$ is the rating value assessed by the expert which satisfies $R_{1}=1$, $R_{2}=2$, …, and $R_{10}=10$; and $m_{j}\left(O_{i}\right)$, $m_{j}\left(S_{i}\right)$ and $m_{j}\left(D_{i}\right)$ are the mass functions of the corresponding rating values assessed by the *i*th expert.

### 3.2. The Improved FMEA Approach Based on the New RPN Model

**Figure 2:**
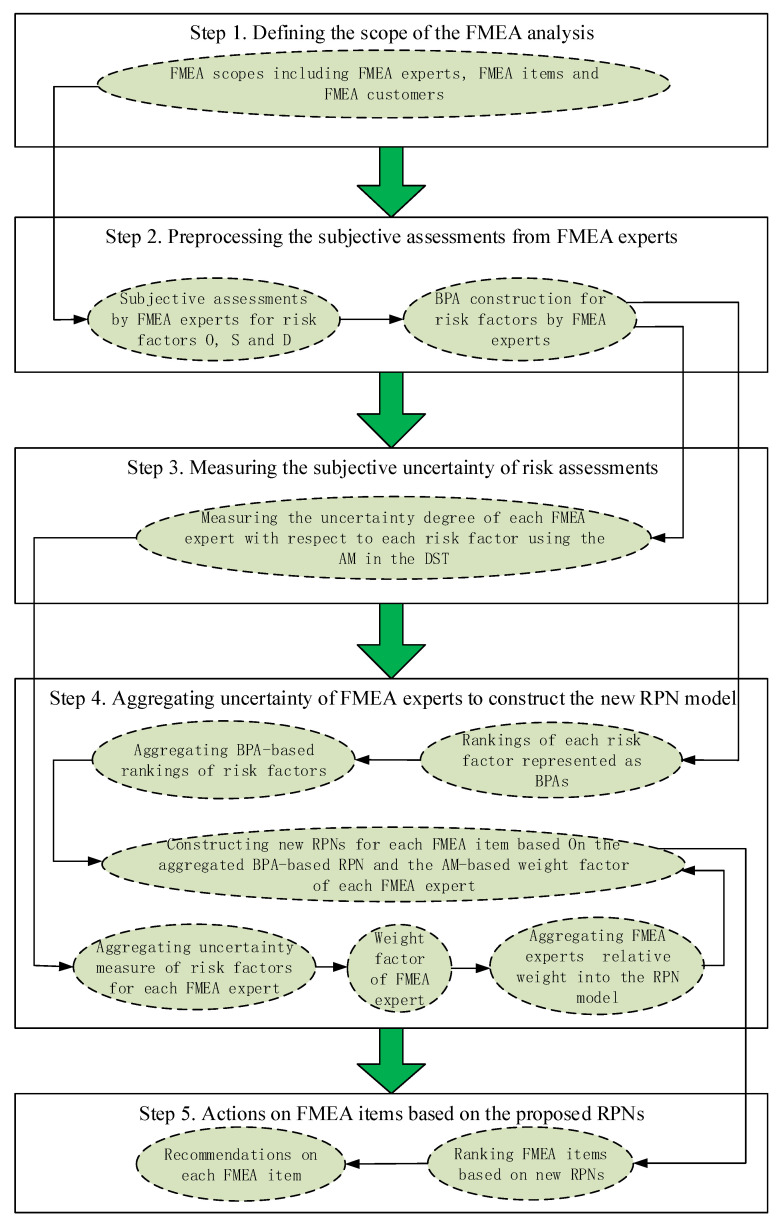
The framework of the new FMEA approach where the ambiguity measure in DST is adopted to measure and aggregate the uncertainty consisted in the assessments of FMEA experts.

The block diagram of the proposed FMEA approach is shown in Figure 2, where the solid arrows indicate the processing of uncertain information and data flow.

Five steps in the improved FMEA approach are shown as follows.

- Step 1. Define the scope of the FMEA analysis.
  The first step of FMEA process is defining the scope of FMEA experts, FMEA items and FMEA customers. FMEA experts should come from different professional groups. The scope of FMEA items should be handled very carefully and defined very cautiously, as well as the customers of FMEA items.
- Step 2. Preprocess the subjective assessments from FMEA experts.
  For each item in the defined FMEA scope, each expert from the FMEA team will give their own assessments. The linguistic assessments on risk factors *O*, *S* and *D* should be constructed as BPA in DST framework for the following processing. Many methods have been adopted to construct BPAs in applications, such as the dynamic BPA method [27], the normal distribution function-based BPA generation method [52], and so on.
- Step 3. Measure the subjective uncertainty of risk assessments.
  The subjective assessments of FMEA experts have been modeled as BPAs according to the previous step. Thus, for each risk factor of each FMEA item, the corresponding uncertain degree can be measured by the AM in DST framework. Equation (8) presents the definition of the uncertainty for each risk factor.
- Step 4. Aggregate uncertainty of FMEA experts to construct the new RPN model.
  The subjective assessments on each risk factor of each FMEA item are expressed as BPAs, thus the rankings of each risk factor represented as BPAs should be aggregated to construct the final ranking of each risk factor for each FMEA item. Equation (9) presents the aggregated BPA-based rankings of each risk factor by each FMEA expert. Simultaneously, the AM-based uncertainty for each FMEA item is aggregated by Equation (7) to construct the weight factor of each FMEA expert. Finally, the new RPN for each FMEA item based on the aggregated BPA-based RPN and the AM-based weight factor of each FMEA expert can be constructed according to Equation (6).
- Step 5. Act on FMEA items based on the proposed RPNs.
  Rankings of FMEA items is based on the new RPN model. The recommendations for all FMEA items are based on the proposed FMEA approach. The FMEA item which has a higher risk level is always more critical, thus it should be handled in advance.

The framework of the proposed FMEA method is consistent with ISO 31000 [56] on risk assessment, e.g., the process of calculating the RPN value in FMEA can be integrated into ISO 31000 as a quantification method of risk evaluation. Some researchers have built a hybrid method for risk identification, evaluation, and control based on FMEA and ISO 31000 [57].

## 4. Application in Fault Evaluation of Aircraft Turbine Rotor Blades

As a key component of aircraft, the possible risk in turbine rotor blades needs cautious study. The case study in [51,52] is adopted to verify the improved FMEA method. Some photos of aircraft blades as well as the sample of failures in aircraft blades can be found in [1,8]. 

Step 1. Define the scope of the FMEA analysis. 

The ways of defining the scope of FMEA analysis is usually based on experience of a group or an organization under a given process or object. Since how to define the scope of a FMEA process is not the concern of this paper, the scope of the FMEA in the case study is adopted from [51] directly.

Step 2. Preprocess the subjective assessments from FMEA experts. 

The BPAs constructed by the new method in [52] are adopted for this step.

Step 3. Measure the subjective uncertainty of risk assessments. 

Take the first failure mode and effect analysis (denoted as fmea1) as an example. The BPAs of fmea1 are shown in Table 1.

For fmea1, the measuring results for risk factors by Expert 1 with Equation (8) is calculated as follows:

(10) $$\begin{array}{c} AM\left(O_{1}\right)=-\sum_{O_{1}\in A\subseteq X}BetP_{m}\left(A\right)\log_{2}\left(BetP_{m}\left(A\right)\right)=0.9710,\\ AM\left(S_{1}\right)=-\sum_{S_{1}\in A\subseteq X}BetP_{m}\left(A\right)\log_{2}\left(BetP_{m}\left(A\right)\right)=0.9219,\\ AM\left(D_{1}\right)=-\sum_{D_{1}\in A\subseteq X}BetP_{m}\left(A\right)\log_{2}\left(BetP_{m}\left(A\right)\right)=0.9219. \end{array}$$

Step 4. Aggregate uncertainty of FMEA experts to construct the new RPN model. 

According to Definition 5 and the AM value calculated in Step, the corresponding weight factor of Expert 1 can be calculated based on Equation (7):

(11) $$w\left(e_{1}\right)=AM\left(O_{1}\right)+AM\left(S_{1}\right)+AM\left(D_{1}\right)=2.8148,$$

Then, the aggregated rating value of risk factors by Expert 1 can be calculated with Equation (9):

(12) $$\begin{array}{c} O_{1}=\sum_{j=1}^{10}R_{j}m_{j}\left(O_{1}\right)=R_{3}m_{3}\left(O_{1}\right)+R_{4}m_{4}\left(O_{1}\right)=3.6000,\\ S_{1}=\sum_{j=1}^{10}R_{j}m_{j}\left(S_{1}\right)=R_{6}m_{6}\left(S_{1}\right)+R_{7}m_{7}\left(S_{1}\right)+R_{8}m_{8}\left(S_{1}\right)=7.0000,\\ D_{1}=\sum_{j=1}^{10}R_{j}m_{j}\left(D_{1}\right)=R_{1}m_{1}\left(D_{1}\right)+R_{2}m_{2}\left(D_{1}\right)+R_{3}m_{3}\left(D_{1}\right)=2.0000. \end{array}$$

Similarly, with Equations (7)–(9), the calculation results of fmea1 by Experts 2 and 3 is shown in Table 2. Thus, according to the definition in Equation (6), the $RPN_{am}$ of fmea1 can be calculated as follows:

(13) $$RPN_{am}=\sum_{i=1}^{n}\frac{w\left(e_{i}\right)}{\sum_{i=1}^{n}w\left(e_{i}\right)}O_{i}S_{i}D_{i}=46.4875.$$

Table 3 shows the calculation results of all 17 failure modes (denoted as fmea1, fmea2, …, fmea17) for the compressor rotor blade in [52] with the proposed method, as well as a comparison with some other methods.

**Figure 3:**
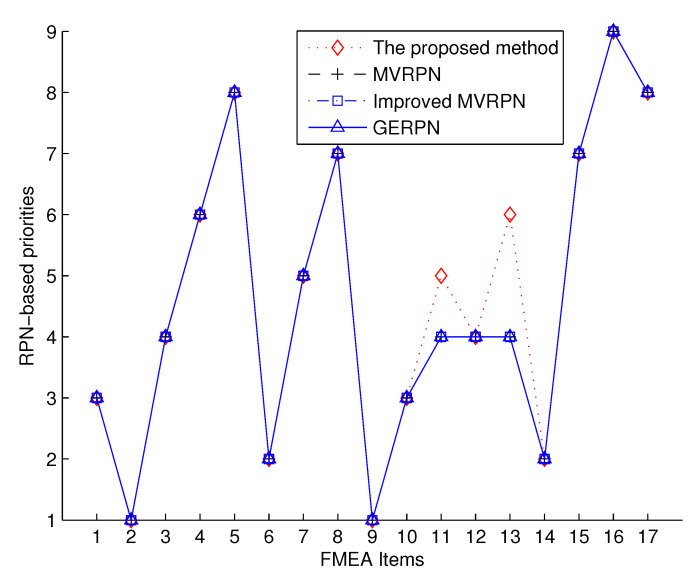
FMEA ranking of the compressor rotor blade (FMEA Items 1–8) and the turbo rotor blade (FMEA Items 9–17) based on the proposed method, as well as the methods in [51] (MVRPN), [52] (the improved MVRPN) and [53] (GERPN).

For the convenience of comparison, the RPN values with other methods are also listed in Table 3. The $RPN_{am}$-based priorities of the compressor rotor blade and the turbo rotor blade are presented in Figure 3.

Step 5. Act on FMEA items based on the proposed RPNs. 

The following actions in FMEA approach are based on the new RPN value-based ranking of all the FMEA items. In general, the smaller is the ranking number, the higher is the risk level. For a process or product, the limited resource should always be adopted to improve the FMEA item with a higher risk level.

For the compressor rotor blade, Figure 3 shows that fmea2 has the highest risk level, while fmea5 has the lowest risk priority level. The $RPN_{am}$-based priorities for the compressor rotor blade are fmea2≻fmea6≻fmea1≻fmea3≻fmea7≻fmea4≻fmea8≻fmea5 (“≻” denotes a higher priority), which is consistent with the methods in [51,52,53]. All FMEA experts have the same belief and ranking assessments on the related FMEA Items fmea3, fmea6 and fmea7. Thus, the RPN values with the proposed method for fmea3, fmea6 and fmea7 are the same as in [51,52], which indicates the efficiency of the new method. Compared with the method in [51], the calculation results for FMEA Items fmea2, fmea4 and fmea8 with $RPN_{am}$ are not integers, indicating that the proposed method is more sensitive in modeling the difference of belief assignment coming from different FMEA experts. This can be a superiority in uncertain information processing because it means more accurate in capturing changes of subjective assessments.

The $RPN_{am}$-based priorities for the turbo rotor blade are fmea9≻fmea14≻fmea10≻fmea12≻fmea11≻fmea13≻fmea15≻fmea17≻fmea16, which is consistent with the methods in [51,52,53] in general. It should be noted that, as shown in Figure 3, the new FMEA approach can figure out all the FMEA items with different ranking values and priorities, while the other methods in [51,52,53] failed to distinguish the difference of rankings and priorities among FMEA Items fmea11, fmea12 and fmea13. The reason exists in the FMEA experts’ assessments on these FMEA items. As shown in [51,52], there is no difference in the assessments coming from different FMEA experts for fmea11, while there are differences for fmea12 and fmea13 regarding the assessments from different FMEA experts, but the methods in [51,52,53] all failed to model and present the difference of different FMEA experts assessments in the final RPN models. The experiment results verify that the new FMEA approach can model the subjective assessments of FMEA experts in a more accurate and reasonable way in some practical cases, which, in fact, is achieved significantly by the new RPN model.

## 5. Conclusions

An improved FMEA approach with a newly defined RPN is proposed in this paper, where the AM in DST is adopted to construct the new RPN model which can measure and aggregate the subjective uncertainty of FMEA experts’ assessments. The proposed method modifies the classical RPN model by expressing the uncertain degree of expert opinions in FMEA as the relative importance of each FMEA expert. Each part of assessment with respect to each risk factor in a FMEA item is modeled as the evidence in DST. The uncertain degree of each piece of evidence is modeled as the ambiguity degree by the AM in DST framework. The relative importance of different subjective assessments in the new RPN comes from the assessment of FMEA experts itself, which is a way of mining the inner uncertainty of subjective assessment. We believe this is a typical idea for modeling uncertainty of subjective assessment in engineering. An application in fault evaluation of aircraft turbine rotor blades verifies the applicable and useful of the new method. The new method is easy to understand by ordinary engineers and technicians, thus it can be easily extended to practical engineering applications. In addition, the new FMEA approach is useful, especially for complicated cases where there is a complex professional FMEA group, thus the relative importance of different experts must be taken into consideration.

Possible future work will focus on the following directions. On the one hand, currently, we only focus on applying the existing uncertainty measure to model the uncertainty of expert opinions. In future research, we will try to propose some new methods for quantification of uncertain information, since there are some new properties and features needed for uncertainty measure in the evidence theory [59]. On the other hand, how to construct belief functions based on expert opinions [60] is a key issue in real applications, thus, how to construct the belief functions based on FMEA experts’ opinions needs further study. Furthermore, the relative importance among each risk factor also needs proper addressing in practical engineering. 

## Figures and Tables

**Table 1:** BPAs of experts’ assessment information for fmea1 (adopted from [52]).

Risk Factor	Expert 1	Expert 2	Expert 3
*O*	$\begin{array}{c} m\left(3\right)=0.4,\\ m\left(4\right)=0.6. \end{array}$	$\begin{array}{c} m\left(3\right)=0.9,\\ m\left(4\right)=0.1. \end{array}$	$\begin{array}{c} m\left(3\right)=0.8,\\ m\left(4\right)=0.2. \end{array}$
*S*	$\begin{array}{c} m\left(6\right)=0.1,\\ m\left(7\right)=0.8,\\ m\left(8\right)=0.1. \end{array}$	$\begin{array}{c} m\left(6\right)=0.1,\\ m\left(7\right)=0.8,\\ m\left(8\right)=0.1. \end{array}$	$\begin{array}{c} m\left(6\right)=0.1,\\ m\left(7\right)=0.8,\\ m\left(8\right)=0.1. \end{array}$
*D*	$\begin{array}{c} m\left(1\right)=0.1,\\ m\left(2\right)=0.8,\\ m\left(3\right)=0.1. \end{array}$	$\begin{array}{c} m\left(1\right)=0.1,\\ m\left(2\right)=0.8,\\ m\left(3\right)=0.1. \end{array}$	$\begin{array}{c} m\left(1\right)=0.1,\\ m\left(2\right)=0.8,\\ m\left(3\right)=0.1. \end{array}$

**Table 2:** AM and aggregated rating values of each expert for fmea1.

fmea1	Expert 1	Expert 2	Expert 3
$AM\left(\cdot \right)$	$\begin{array}{c} AM\left(O_{1}\right)=0.9710\\ AM\left(S_{1}\right)=0.9219\\ AM\left(D_{1}\right)=0.9219 \end{array}$	$\begin{array}{c} AM\left(O_{2}\right)=0.4690\\ AM\left(S_{2}\right)=0.9219\\ AM\left(D_{2}\right)=0.9219 \end{array}$	$\begin{array}{c} AM\left(O_{3}\right)=0.4507\\ AM\left(S_{3}\right)=0.9219\\ AM\left(D_{3}\right)=0.9219 \end{array}$
Rating	$\begin{array}{c} O_{1}=3.6000\\ S_{1}=7.0000\\ D_{1}=2.0000 \end{array}$	$\begin{array}{c} O_{2}=3.1000\\ S_{2}=7.0000\\ D_{2}=2.0000 \end{array}$	$\begin{array}{c} O_{3}=3.2000\\ S_{3}=7.0000\\ D_{3}=2.0000 \end{array}$
$w\left(e_{i}\right)$	2.8148	2.3129	2.2946

**Table 3:** A comparison of RPN values.

FMEA Item	The New Method	MVRPN [51]	Improved MVRPN [52]	GERPN [53]
fmea1	46.4875	42.56	42.56	3.4910
fmea2	64.7921	64.00	64.05	3.9994
fmea3	30.0000	30.00	30.00	3.1069
fmea4	17.5822	18.00	17.97	2.6205
fmea5	3.6671	4.17	3.14	1.6095
fmea6	60.0000	60.00	60.00	3.9143
fmea7	21.0000	21.00	21.00	2.7586
fmea8	16.2000	15.00	15.00	2.4660
fmea9	70.5947	78.92	79.57	4.2881
fmea10	60.0000	60.00	60.00	3.9143
fmea11	50.0000	50.00	50.00	3.6836
fmea12	53.8039	50.00	50.00	3.6836
fmea13	49.3333	50.00	50.00	3.6836
fmea14	60.6337	60.00	60.04	3.9143
fmea15	41.9161	42.00	42.09	3.4756
fmea16	21.2967	23.88	23.86	2.8794
fmea17	31.2810	30.05	30.05	3.1089
